# Supplementary material for: Risk of carotid plaques according to triglyceride-glucose index stratified by thyroid function: A cross-sectional study
Source: PLoS One. 2022 Dec 30;17(12):e0279494. doi: 10.1371/journal.pone.0279494 (PMC9803248; doi:10.1371/journal.pone.0279494)
Supplement: Attachment — Submitted filename: Recommendation.docx [file pone.0279494.s002.docx]

This study assessed the risk of carotid plaques according to TyG index and thyroid function status in the general population. This work is important and well-structured and the results support the authors ‘conclusions. Statistical procedures seemed steady. Confounding factors were excluded to conduct their results. These are the strengths of their study. I have some remarks aimed at improving the readability and presentation of the data.

1. The type of study (cross-sectional) is better to be mentioned in the title.
2. Inclusion and exclusion criteria should be added.
3. Please clarify statistical analysis section in more detail. For example, method of controlling confounders in this study.
4. In discussion section, although the results of the present study have been well compared with the findings of previous studies, the reason for discrepancy between the results of the current study and previous works should be discussed a little.
5. Authors need to add the strengths of this study before the paragraph explaining study limitations.
6. Please check the concordance of the tenses used throughout the text.
