# Supplementary material for: Risk of carotid plaques according to triglyceride-glucose index stratified by thyroid function: A cross-sectional study
Source: PLoS One. 2022 Dec 30;17(12):e0279494. doi: 10.1371/journal.pone.0279494 (PMC9803248; doi:10.1371/journal.pone.0279494)
Supplement: Attachment — Submitted filename: Respose to Reviewers.docx [file pone.0279494.s003.docx]

**Response to the Reviewer #1’s comments**

We would like to express our sincere thanks for the careful review and helpful comments that have been of great assistance in improving this manuscript.

**1. The type of study (cross-sectional) is better to be mentioned in the title.**

**Answer)** Thank you for your valuable comments. We corrected the title as below.

**Corrections)** Title

Risk of Carotid Plaques According to Triglyceride-Glucose Index Stratified by Thyroid Function: a Cross-sectional Study

**2. Inclusion and exclusion criteria should be added.**

**Answer)** All participants who underwent carotid ultrasound underwent a health examination consisting of a series of checkup items including blood laboratory tests. And, if there were any items that were not written in the self-report questionnaire, they were asked to fill out the questionnaire again. So there were no participants to be excluded due to missing data. In addition, variables with high correlation with carotid plaque, such as hypertension or diabetes, were not excluded and were adjusted as confounding factors. However, because the study subject was limited to subclinical thyroid dysfunction and normal thyroid function, cases with abnormal fT4 results were excluded. We described the inclusion and exclusion criteria in the original manuscript as below.

**In the original manuscript)** Subject and methods section, Page 5, Line 55-60

This study retrospectively reviewed the data of 2,931 individuals who underwent carotid ultrasound as part of a comprehensive health examination at the Health Promotion Center of Soonchunhyang University Hospital between January 2016 and June 2018. Among these participants, 28 were excluded for abnormal free thyroxine (fT4) levels [fT4 <0.89 ng/dL (n=9) and fT4 >1.78 ng/dL (n=19)]. Finally, 2,903 subjects were eligible for analysis.

**3. Please clarify statistical analysis section in more detail. For example, method of controlling confounders in this study.**

**Answer)** Thank you for helpful comments. As you noted, the confounding variables were described in more detail as follows.

**Corrections)** Subject and methods section, Page 8, Line 137-141

Additional adjustments were made for confounding variables, such as sex, age (years), smoking (current smoker, ex-smoker or never smoker), obesity (BMI ≥25 kg/m2 or BMI <25 kg/m2), hypertension (BP ≥140/90 mmHg/antihypertensive medication or no) and diabetes mellitus (HbA1c ≥6.5%/antidiabetic medication or no).

**4. In discussion section, although the results of the present study have been well compared with the findings of previous studies, the reason for discrepancy between the results of the current study and previous works should be discussed a little.**

**Answer)** Thank you for the valuable comments. In discussion section, we described the region and population in which the study was conducted and corrected incomplete part about the reasons for discrepancy among studies.

**Corrections)** Discussion section, Page 18~19, Line 288-313

Therefore, we further investigated the risk of carotid plaques according to the TyG index, stratified by thyroid function status. The proportion of subjects with significant carotid plaques was significantly different according to insulin resistance and subclinical thyroid dysfunction. Subjects in the HiTyG-SHypo group had increased odds of the significant carotid plaques compared to those in the LoTyG-Eu group. This relationship remained statistically significant after adjusting for the major atherosclerotic risk factors.

Several studies have attempted to link subclinical thyroid dysfunction with carotid plaques [21-24]. A small case-control study in Macedonia reported that subclinical hypothyroidism was associated with carotid plaques, independent of the risk factors for atherosclerosis [24], which supports some of our data. A population-based study in Germany demonstrated that subjects with decreased serum TSH levels had increased odds for carotid plaques compared to those with normal serum TSH levels, but there was no associations between elevated serum TSH levels and carotid plaques [23]. Another population-based cross-sectional study in Italy found no correlation between subclinical thyroid dysfunction and carotid plaques [21]. Recently, Kim et al. examined the association between subclinical thyroid dysfunction and carotid plaques in a cross-sectional and longitudinal assessment of Korean healthy individuals [22]. At baseline, carotid plaques were more prevalent in the subclinical hypothyroidism group than in the euthyroidism group [22]. However, the effect of subclinical hypothyroidism on the cumulative incidence of new carotid plaques was not significantly different with follow-up time [22]. These inconsistent findings [21-24] are possibly due to regional differences in iodine intake, which may affect thyroid function, and differences in the study design and study population. In addition, since insulin resistance was not considered in previous studies [21-24], additional studies to validate our findings are needed to determine whether the risk of carotid plaques differs according to subclinical thyroid dysfunction.

**5. Authors need to add the strengths of this study before the paragraph explaining study limitations.**

**Answer)** Regarding the strength of our study, we described as below. Thank you for valuable comments.

**Corrections)** Discussion section, Page 19, Line 329-330

Despite the strength of large samples and control of extensive data on insulin resistance and several potential confounding factors, this study has some limitations.

**6. Please check the concordance of the tenses used throughout the text.**

**Answer)** Since our native language is not English, the manuscript has been proofread by a native English speaker and the certificate is attached as a supplementary file. Thank you very much.

**Response to the Reviewer #2’s comments**

We would like to express our sincere thanks for the careful review and helpful comments that have been of great assistance in improving this manuscript.

**1. In the introduction section, the last sentence of the third paragraph, it is mentioned that there are few studies in the literature on this topic discussed by the authors in the present study. Please provide reference for this sentence.**

**Answer)** To date, no papers have considered both TyG index and thyroid function. I used the word 'few' to emphasize 'no', but I realized that the English expression was wrong and corrected as below. Thank you for detailed comments.

**Corrections)** Introduction section, Page 4, Line 47-49

To the best of our knowledge, to date, no studies have addressed the differences in the risk of carotid plaques according to the TyG index and thyroid function status.

**2. Table 1 is very crowded and not easily understood as it is. It would be more appropriate to present Table 1 as two new tables according to the low and high TyG index. Also, post-hoc analyzes should be made for the pairwise comparisons after Kruskal-Wallis tests and this statistical data added to the table footnotes.**

**Answer)** Thank you for helpful comments. The table 1 was very crowded because it not only compared the low TyG and high TyG groups as a whole, but also analyzed according to thyroid function status in each TyG index group. So, Table 1 was changed to show the comparison between low TyG and high TyG groups, and Table 2 shows the comparison according to thyroid function status in each TyG index group. In addition, post-hoc analyses using Tukey’s multiple comparison test were made and added to the table footnotes.

**Corrections)** Table 1 and Table 2

**Table 1.** Baseline characteristics of participants with respect to the triglyceride-glucose index.

| Variables | Low TyG index (n=1457) | High TyG index (n=1446) | Overall | p value |
| --- | --- | --- | --- | --- |
| Age (years) | 51.0 (43.0, 57.0) | 53.0 (74.0, 58.0) | 52.0 (46.0, 58.0) | <0.001 |
| Sex (male), n (%) | 946 (65%) | 1267 (88%) | 2213 (76%) | <0.001 |
| Current smoker, n (%) | 244 (17%) | 420 (29%) | 664 (23%) | <0.001 |
| Hypertension, n (%)^1)^ | 273 (19%) | 445 (31%) | 718 (25%) | <0.001 |
| Diabetes mellitus, n (%)^2)^ | 56 (4%) | 249 (18%) | 305 (10%) | <0.001 |
| BMI (kg/m^2^)  Obesity, n (%)^3)^ | 23.4 (21.5, 25.2)  398 (27%) | 25.5 (23.8, 27.5)  833 (58%) | 24.4 (22.6, 26.5)  1231 (42%) | <0.001  <0.001 |
| Systolic BP (mmHg) | 119 (109, 129) | 126 (116, 135) | 123 (112, 132) | <0.001 |
| Diastolic BP (mmHg) | 73 (66, 80) | 78 (71, 85) | 75 (69, 82) | <0.001 |
| Total cholesterol (mg/dL) | 185 (161, 207) | 200 (177, 224) | 192 (168, 215) | <0.001 |
| HDL cholesterol (mg/dL)  Male  Female | 58.0 (50.0, 67.0)  68.0 (59.0, 79.0) | 48.0 (41.0, 55.0)  53.0 (45.0, 63.0) | 52.0 (44.0, 61.0)  64.0 (54.0, 76.0) | <0.001  <0.001 |
| Triglycerides (mg/dL) | 84 (66, 102) | 170 (137, 219) | 117 (84, 169) | <0.001 |
| Fasting glucose (mg/dL) | 88 (83, 94) | 97 (90, 108) | 92 (86, 101) | <0.001 |
| HbA1c (%) | 5.4 (5.2, 5.6) | 5.6 (5.3, 6.1) | 5.5 (5.2, 5.8) | <0.001 |
| TyG index^4)^ | 8.23 (7.98, 8.44) | 9.05 (8.81, 9.33) | 8.62 (5.24, 9.04) | <0.001 |
| TSH (mIU/L) | 2.06 (1.39, 3.05) | 1.99 (1.38, 2.83) | 2.03 (1.38, 2.98) | 0.113 |
| fT4 (ng/dL) | 1.30 (1.18, 1.42) | 1.32 (1.20, 1.42) | 1.31 (1.19, 1.42) | 0.089 |
| CRP (mg/L) | 0.04 (0.02, 0.08) | 0.07 (0.04, 1.14) | 0.05 (0.03, 1.11) | <0.001 |
| Carotid IMT (mm)^5)^ | 0.54 (0.48, 0.61) | 0.58 (0.52, 0.65) | 0.56 (0.50, 0.63) | <0.001 |
| Carotid plaque score^6)^ | 2.0 (0.0, 2.0) | 2.0 (1.0, 3.0) | 2.0 (1.0, 3.0) | <0.001 |

**Table 2.** Clinical and biochemical characteristics of participants according to thyroid function status.

| Variables | Low TyG index (n=1457) | | | |
| --- | --- | --- | --- | --- |
|  | Subclinical hyperthyroidism  (n=24) | Euthyroidism  (n=1248) | Subclinical hypothyroidism  (n=185) | p value |
| Age (years) | 54.0 (47.0, 56.0)^ac^ | 51.0 (43.0, 57.0)^a^ | 53.0 (47.0, 59.0)^bc^ | 0.001 |
| Sex (male), n (%) | 8 (33%) | 844 (68%) | 94 (51%) | <0.001 |
| Current smoker, n (%) | 3 (13%) | 224 (18%) | 17 (9%) | 0.003 |
| Hypertension, n (%)^1)^ | 2 (8%) | 233 (19%) | 38 (21%) | 0.349 |
| Diabetes mellitus, n (%)^2)^ | 0 (0%) | 46 (4%) | 10 (5%) | 0.322 |
| BMI (kg/m^2^)  Obesity, n (%)^3)^ | 23.9 (21.1, 25.0)  5 (21%) | 23.4 (21.6, 25.2)  342 (27%) | 23.3 (21.2, 25.1)  51 (28%) | 0.735  0.772 |
| Systolic BP (mmHg) | 121 (111, 128) | 119 (110, 129) | 118 (108, 128) | 0.220 |
| Diastolic BP (mmHg) | 71 (67, 76) | 73 (66, 80) | 72 (66, 78) | 0.177 |
| Total cholesterol (mg/dL) | 184 (163, 193) | 185 (161, 207) | 186 (161, 215) | 0.225 |
| HDL cholesterol (mg/dL)  Male  Female | 54.0 (48.3, 62.0)  60.0 (51.8, 73.0) | 58.0 (50.0, 67.8)  69.0 (59.0, 80.0) | 56.5 (49.8, 64.3)  67.0 (60.0, 77.0) | 0.357  0.209 |
| Triglycerides (mg/dL) | 83 (71, 104) | 84 (66, 102) | 82 (67, 100) | 0.982 |
| Fasting glucose (mg/dL) | 90 (85, 94) | 88 (83, 94) | 88 (82, 93) | 0.707 |
| HbA1c (%) | 5.3 (5.2, 5.5) | 5.4 (5.2, 5.6) | 5.4 (5.2, 5.6) | 0.102 |
| TyG index^4)^ | 8.18 (8.10, 8.34) | 8.24 (7.98, 8.45) | 8.20 (7.96, 8.42) | 0.930 |
| TSH (mIU/L) | 0.05 (0.04, 0.16)^a^ | 1.90 (1.35, 2.61)^b^ | 5.04 (4.38, 5.83)^c^ | <0.001 |
| fT4 (ng/dL) | 1.62 (1.40, 1.72)^a^ | 1.30 (1.18, 1.41)^b^ | 1.27 (1.14, 1.39)^c^ | <0.001 |
| CRP (mg/L) | 0.4 (0.3, 1.0) | 0.4 (0.2, 0.8) | 0.4 (0.2, 0.7) | 0.705 |
| Carotid IMT (mm)^5)^ | 0.54 (0.47, 0.60) | 0.54 (0.48, 0.61) | 0.54 (0.49, 0.62) | 0.747 |
| Carotid plaque score^6)^ | 2.0 (0.0, 2.0)^ac^ | 2.0 (0.0, 2.0)^a^ | 2.0 (1.0, 3.0)^bc^ | 0.045 |

(A) Low TyG index.

| Variables | High TyG index (n=1446) | | | |
| --- | --- | --- | --- | --- |
|  | Subclinical hyperthyroidism  (n=19) | Euthyroidism  (n=1267) | Subclinical hypothyroidism  (n=160) | p value |
| Age (years) | 50.0 (44.8, 55.3) | 53.0 (47.0, 58.0) | 54.0 (47.0, 60.0) | 0.293 |
| Sex (male), n (%) | 17 (89%) | 1121 (88%) | 129 (81%) | 0.017 |
| Current smoker, n (%) | 3 (16%) | 386 (30%) | 31 (20%) | 0.031 |
| Hypertension, n (%)^1)^ | 7 (37%) | 395 (31%) | 43 (27%) | 0.457 |
| Diabetes mellitus, n (%)^2)^ | 3 (16%) | 224 (18%) | 22 (14%) | 0.457 |
| BMI (kg/m^2^)  Obesity, n (%)^3)^ | 24.9 (23.5, 28.4)  9 (47%) | 25.5 (23.9, 27.5)  735 (58%) | 25.6 (23.6, 27.6)  89 (56%) | 0.858  0.561 |
| Systolic BP (mmHg) | 120 (116, 135) | 126 (116, 135) | 127 (117, 135) | 0.719 |
| Diastolic BP (mmHg) | 75 (67, 83) | 78 (71, 85) | 79 (70, 87) | 0.394 |
| Total cholesterol (mg/dL) | 196 (167, 208)^ac^ | 199 (176, 223)^a^ | 207 (186, 231)^bc^ | 0.020 |
| HDL cholesterol (mg/dL)  Male  Female | 49.0 (37.5, 56.0)  46.5 (38.0, 55.0) | 48.0 (41.0, 55.0)  53.0 (46.0, 62.0) | 48.0 (41.0, 56.0)  50.0 (45.5, 67.5) | 0.992  0.630 |
| Triglycerides (mg/dL) | 162 (139, 250) | 169 (136, 216) | 174 (147, 227) | 0.095 |
| Fasting glucose (mg/dL) | 97 (90, 113) | 98 (90, 108) | 96 (90, 109) | 0.523 |
| HbA1c (%) | 5.9 (5.3, 6.1) | 5.6 (5.3, 6.1) | 5.6 (5.3, 6.0) | 0.639 |
| TyG index^4)^ | 8.95 (8.75, 9.47) | 9.04 (8.80, 9.31) | 9.07 (8.84, 9.40) | 0.266 |
| TSH (mIU/L) | 0.08 (0.05, 0.12)^a^ | 1.84 (1.32, 2.51)^b^ | 5.11 (4.44, 6.57)^c^ | <0.001 |
| fT4 (ng/dL) | 1.50 (1.43, 1.63)^a^ | 1.32 (1.21, 1.42)^b^ | 1.27 (1.13, 1.41)^c^ | <0.001 |
| CRP (mg/L) | 0.8 (0.5, 2.6) | 0.7 (0.4, 1.4) | 0.7 (0.3, 1.4) | 0.499 |
| Carotid IMT (mm)^5)^ | 0.57 (0.55, 0.65) | 0.58 (0.52, 0.65) | 0.58 (0.52, 0.65) | 0.959 |
| Carotid plaque score^6)^ | 2.0 (1.0, 3.0) | 2.0 (1.0, 3.0) | 2.0 (1.0, 3.0) | 0.834 |

(B) High TyG index.

^a,b,c^The same letters indicate non-significant difference between groups based on Tukey’s multiple comparison test.

**3. According to current literature data, it is clear that high triglyceride-glucose index, presence of subclinical hypothyroidism and insulin resistance increase cardiometabolic risk. Although this study is original and comprehensive in terms of methodology and design, it is clear that predictable results accordance with the literature will be obtained.**

**Answer)** We totally agree with your comments. Thank you very much for the detailed review and thoughtful comments.
